# Effect of Nutritional Interventions on Micronutrient Status in Pregnant Malawian Women with Moderate Malnutrition: A Randomized, Controlled Trial

**DOI:** 10.3390/nu10070879

**Published:** 2018-07-07

**Authors:** Cambria M. Glosz, Andrew A. Schaffner, Scott K. Reaves, Mark J. Manary, Peggy C. Papathakis

**Affiliations:** 1 Department of Food Science and Nutrition, California Polytechnic State University, San Luis Obispo, CA 93407, USA; Cambriaglosz@gmail.com (C.M.G.); aschaffn@calpoly.edu (A.A.S.); sreaves@calpoly.edu (S.K.R.); 2 Washington University School of Medicine, Washington University, St. Louis, MO 63110, USA; manary@kids.wustl.edu

**Keywords:** maternal malnutrition, micronutrient deficiency, supplementary foods, pregnancy, Malawi

## Abstract

Micronutrient deficiencies during pregnancy are common in Africa and can cause adverse outcomes. The objective was to measure micronutrient status and change in moderately malnourished pregnant Malawian women randomized to one of three nutritional interventions. Serum vitamin B_12_, 25-hydroxyvitamin D, folate, retinol, ferritin, zinc, albumin and C-reactive protein were measured in pregnant women with MUAC ≥20.6 cm and ≤23.0 cm at enrollment (*n* = 343) and after 10 weeks (*n* = 229) of receiving: (1) ready-to-use supplementary food (RUSF); (2) fortified corn-soy blend (CSB+) with multiple-micronutrient supplement (CSB+UNIMMAP); or (3) CSB+ with iron and folic acid (CSB+IFA). Each provided 100–300% Recommended Dietary Allowance of most micronutrients and 900 kcal/day. Birth length was measured in 272 infants. Enrollment measurements indicated deficiencies in vitamin B_12_ (20.9%) and zinc (22.3%), low values of ferritin (25.1%) and albumin (33.7%), and elevated CRP (46.0%). Vitamin B_12_ is known to decrease in the third trimester; the RUSF group had the smallest decrease from enrollment to week 10 (3%), compared to 20% decrease in the CSB+IFA group and 8% decrease in the CSB+UNIMMAP group (*p* = 0.001). Mean serum 25-hydroxyvitamin D increased most in the RUSF group (+6.4 ng/mL), compared to CSB+IFA (+1.7 ng/mL) and CSB+UNIMMAP (+2.7 ng/mL) (*p* < 0.001). Micronutrient deficiencies and inflammation are common among moderately malnourished pregnant women and had little improvement despite supplementation above the RDA, with the exception of vitamins B_12_ and D.

## 1. Introduction

Micronutrient malnutrition is widespread in sub-Saharan Africa, with pregnant women being particularly vulnerable to becoming undernourished due to the higher nutrient requirements necessary to sustain the mother and the fetus [1]. An environment of poverty and food shortages results in the increased prevalence of maternal malnutrition, leading to short- and long-term health consequences to both a mother and her child [1,2].

Deficiencies in vitamins A, D, B_12_, and folate and minerals iron and zinc during pregnancy can lead to a myriad of negative maternal consequences, including night blindness, anemia, increased susceptibility to infections, poor wound healing, and maternal mortality. Infant-related consequences include small for gestational age (SGA) and low birth weight (LBW) newborns, increased risk of premature birth and stillbirth, neural tube defects and congenital abnormalities, and decreased cognitive and motor development [2,3,4,5,6,7,8].

The current micronutrient standard of care for pregnant women in most developing countries is supplementation with iron and folic acid (IFA). Recent research has questioned whether a multiple micronutrient supplement (MMN) would be more beneficial to maternal and infant nutrition than IFA alone [8]. Ready-to-use supplementary foods (RUSF) typically consist of an emulsion of protein, lipids and micronutrients and are used to treat moderate malnutrition in young children and may benefit malnourished adults [9]. A small-quantity lipid-based supplement given to pregnant women without malnutrition in Malawi resulted in a 0.2 mm increase in infant birth mid-upper arm circumference (MUAC) compared to those receiving IFA supplements alone [10]. In Ghana, infants of women receiving a lipid-nutrient supplement had greater birth weights than those in the MMN and IFA groups [11]. Few maternal supplementation trials have documented micronutrient status prior to and after nutritional intervention.

Mamachiponde was a randomized controlled trial that measured the effectiveness of a peanut- and dairy-based RUSF against two other nutritional interventions: (1) MMN supplement plus a fortified corn-soy blended flour (CSB+UNIMMAP) or (2) IFA supplement plus fortified corn-soy blended flour (CSB+IFA). The present sub-study of Mamachiponde aimed to assess the prevalence of deficiency and mean serum or plasma concentrations of vitamins A, D, B_12_ and folate, minerals zinc and iron, and proteins albumin and C-reactive protein in a sample of moderately malnourished pregnant Malawian women before and ten weeks after receiving one of the three interventions.

## 2. Materials and Methods

This sub-study was an investigator-blinded, randomized controlled clinical trial designed to determine if there were any differences in serum or plasma micronutrient concentrations between groups after receiving RUSF, CSB+UNIMMAP, or CSB+IFA for 10 weeks. The sub-study was done in the context of a larger clinical trial, named the Mamachiponde study, conducted to compare clinical outcomes with different supplementary foods [12]. The primary outcomes of Mamachiponde were maternal recovery from malnutrition, change in maternal MUAC, newborn birth weight and length, and prematurity. Secondary outcomes of Mamachiponde included change in maternal weight, change in maternal hemoglobin concentrations, duration of treatment, and infant anthropometrics and survival at 6 and 12 weeks.

The sub-study was conducted between September 2014 and May 2015 at 15 antenatal clinics in southern Malawi, 12 of which were located in rural areas. Participants were sampled from the Blantyre, Chikhwawa, Mulanje and Zomba districts. Pregnant women were recruited into the Mamachiponde study from antenatal clinics if they met the inclusion criteria of a MUAC ≥20.6 cm and ≤23.0 cm and willingness to attend the antenatal clinic every two weeks throughout their pregnancy. Exclusion criteria were women under age 16, those with pregnancy complications, or severe anemia (hemoglobin <70 g/L). In the first six months of the study, women most commonly delivered with a fundal height near 32 cm, therefore, additional inclusion criteria for the sub-study included a fundal height measurement of <22 cm to increase the likelihood that the participant would complete 10 weeks of intervention before delivery. Fundal height was used as a proxy for gestational age. The blood draw sub-study was offered to every woman eligible and enrolled in Mamachiponde. One clinic location had women unwilling to draw blood due to cultural reasons, thus that clinic was dropped from the sub-study. All women provided written and verbal consent via signature or thumbprint for enrollment into both Mamachiponde and the sub-study. This study is registered in ClinicalTrials.gov NCT02120599 and was approved by the Institutional Review Boards at Washington University (St. Louis), Cal Poly (San Luis Obispo), and College of Medicine at University of Malawi.

The primary Mamachiponde trial and primary results are described elsewhere [12]. Briefly, participants were randomly assigned to one of three treatment groups using a random number generator, which assigned identification numbers into three treatment groups in blocks of 60; women chose a sealed envelope containing a study identification number. All newly enrolled women were invited to participate in the sub-study until 343 women were enrolled. The control group (CSB+IFA) received 5 kg fortified CSB+ every two weeks plus 60 mg iron and 400 µg folic acid daily (Table 1). The CSB+UNIMMAP treatment included 5 kg fortified CSB+ every two weeks plus a daily standard antenatal 15-micronutrient tablet (UNIMMAP), which provided a similar amount of energy and protein as the RUSF treatment, with some differences in micronutrient levels. The RUSF intervention provided approximately 900 kcal/day, 36 g protein/day, 136–341% of the Recommended Dietary Allowance (RDA) daily for micronutrients during pregnancy, and essential fatty acids (Table 1). At each bi-weekly visit, compliance was self-reported and adherence to the daily ration was encouraged. In a separate adherence sub-study, a home visit interview was conducted by a study nurse that included questions on frequency of supplement consumption, acceptability, and sharing with family members. The amount of remaining study food was observed.

Blood samples were taken on two occasions from each eligible and willing subject. The first blood draw, noted as week 0, was at enrollment into the Mamachiponde study; the second was 10 weeks later. Venous blood samples were drawn in the morning using Universal Precautions with 21 gauge ×1” blood collection needles (Jelco; Smiths Medical) into serum separator tubes (“Tiger Top” BD Vacutainer tubes; 16 × 100 mm; draw volume capacity of 7.5 mL) for ferritin, vitamins B_12_, A, D, folate, albumin and C-reactive protein (CRP), and into Trace Element heparin tubes for plasma zinc (dark blue BD Vacutainer; 13 × 100 mm; draw volume capacity of 6.0 mL). Total blood drawn was approximately 9–10 mL from each subject at each time (3–4 mL into the Trace Element Vacutainer and 6 mL into the “Tiger Top” Tubes), allowing for 4–5 mL of serum/plasma to be analyzed. Deficiency and marginal status cut-off values utilized are described in Table 2.

After a study nurse drew the blood, tubes were labeled with a barcode number corresponding to the subject’s study ID number, covered with foil to protect from light, and packed in a small cooler box with ice packs for transport. The tubes were centrifuged at 3500 rpm for 10 min at <25 °C (Eppendorf Centrifuge 5804R, Eppendorf; Hamburg, Germany) and the serum/plasma was pipetted into separate storage tubes at the College of Medicine in Blantyre, Malawi. Two mL of serum were pipetted into two separate amber-colored storage tubes for analysis of vitamin B_12_, vitamin D, folate, retinol, ferritin, and proteins albumin and CRP; 1 mL of plasma was pipetted into a clear storage tube for zinc analysis. The tubes were stored at −80 °C. The samples were shipped back to the United States periodically in nitrogen gas tanks where they were analyzed at one of three locations: ferritin, vitamin B_12_, folate, albumin and CRP at Central Coast Pathology laboratory in San Luis Obispo, CA, USA; retinol and 25-hydroxyvitamin D at Physician’s Automated Laboratory in Bakersfield, CA, USA, and zinc at Clinical Pathology Lab in Texas, USA, using the methods stated in Supplementary Materials Table S1.

The original sample size was projected to be 300 women total (100 per treatment group), to detect at least a 0.5 standard deviation unit difference between groups for all nutrients, with 0.05 level of significance and 80% power. The sample size increased during the study period from 300 to 343 to account for higher rates of loss to follow-up than anticipated. Enrollment characteristics and differences between groups were tested for significance using the chi-squared test for categorical variables and ANOVA for continuous variables. For longitudinal measures, the groups were compared using repeated measures analysis of variance (RMANOVA) to analyze mean change in nutrients from week 0 to week 10, controlling for enrollment MUAC, HIV status, parity, and fundal height. Additionally, inflammation via CRP was included for retinol, ferritin, zinc, and albumin. Tukey–Kramer adjustments were performed to identify differences between groups. *p*-values were attained by RMANOVA and partial F-tests for treatment effect (main effect or time by treatment interaction). Normality was assessed by analysis of histograms of residuals and goodness-of-fit tests. To achieve normality, vitamin B_12_, ferritin, and CRP were transformed logarithmically. Geomeans were reported for nutrients that were log transformed. Percent CRP-adjusted deficiency rates were compared using chi-square testing. Outliers that were biologically implausible were removed for zinc (*n* = 1) and CRP (*n* = 1). Statistical analyses were performed using SAS JMP Pro Version 12.1.0 from SAS Institute Inc. (Cary, NC, USA).

## 3. Results

A total of 343 women were enrolled into this sub-study. Eight samples were not included due to physical loss, hemolysis of blood, or inadequate amount of serum/plasma (Supplementary Materials Figure S1). The primary reason that subjects did not complete the study was due to delivery before the second blood draw date (*n* = 62). The participants ranged from 16 to 38 years, with similar demographic and physical characteristics, except that fundal height was about 1 cm smaller in the RUSF group at enrollment (*p* = 0.04, Table 3). There were no differences in demographics or physical characteristics between women in the primary Mamachiponde study and women in the sub-study (data not shown). Compliance was good overall, with no differences between the treatment groups with either food or supplement adherence. Over-adherence to food was common in all groups, in that women were consuming more of the food ration earlier in the allotted two weeks.

Deficiencies in multiple micronutrients were seen at enrollment. For the entire study population, 20.9% were deficient in vitamin B_12_, 21.5% and 35.2% were either marginal or deficient for folate and vitamin D, respectively. When using inflammation cut-offs, 32.4% were marginal or deficient for retinol and 25.1% were deficient for ferritin. Using trimester cut-off adjustment for zinc, 22.3% were considered deficient (Table 4). Elevated C-reactive protein (CRP) at enrollment was high at 46%.

Upon enrollment, nutrient concentrations were not significantly different across treatment groups (Table 5). Vitamin B_12_ was lower at week 10 compared to vitamin B_12_ at enrollment in all treatment groups, as is expected during pregnancy [13]. The RUSF group experienced the smallest reduction in serum vitamin B_12_ throughout treatment, as noted by the multiplicative change of 0.97 (indicating 3% decrease from enrollment to week 10), while the CSB+IFA and CSB+UNIMMAP groups had a decrease in serum vitamin B_12_ concentrations of 20% and 8%, respectively (*p* = 0.001; Table 5). Tukey-Kramer adjustments indicate differences in vitamin B_12_ concentrations at week 10 between RUSF and CSB+IFA groups and between CSB+IFA and CSB+UNIMMAP; there was no difference between the RUSF and CSB+UNIMMAP groups (Table 5). The CSB+IFA group experienced the greatest increase in percent vitamin B_12_ deficient; the RUSF group had a significantly lower percent deficient in vitamin B_12_ from enrollment to week 10 (*p* <0.001, Table 4). The CSB+UNIMMAP treatment provided the most vitamin B_12_ (7.3 µg; 280% RDA), compared to 4.7 µg and 5.5 µg in the CSB+IFA and RUSF foods, respectively (Table 1).

Serum vitamin D improved in all groups, but was significantly greater at week 10 in the RUSF group compared to CSB+IFA and CSB+UNIMMAP groups (Table 5). The RUSF group experienced an increase of 6.4 ng/mL from enrollment to week 10, compared to an increase of 1.7 and 2.7 ng/mL in the CSB+IFA and CSB+UNIMMAP groups, respectively (*p* <0.001). Tukey comparisons indicated differences in vitamin D at week 10 between RUSF and both CSB+IFA and CSB+UNIMMAP (Table 5). Vitamin D insufficiency was reduced in all treatment groups by >8 percentage points, but this reduction was not significantly different by treatment group (Table 4). The RUSF supplementary food contained 16–33% more vitamin D, at 30 µg vitamin D per day (200% RDA), compared to 20 µg and 25 µg in the CSB+UNIMMAP and CSB+IFA foods, respectively. At enrollment, women with a 1 ng/mL greater vitamin D concentration were further along in their pregnancies by 0.24 weeks (*p* = 0.02). Interestingly, women who were HIV-positive had a higher mean vitamin D at enrollment (37.8 ng/mL), when controlling for CRP and clinic site, compared to HIV-negative women (32.8 ng/mL, *p* < 0.01). Vitamin D concentrations were highest in those from the Ngabu clinic (Chikhwawa district) (40.4 ng/mL), compared to the lowest mean levels in those from the Makwapala clinic (Zomba district) (30.9 ng/mL) (*p* < 0.01).

Serum folate increased throughout treatment in all groups, and was highest in the CSB+IFA group at week 10, although this was not significantly different from the other two groups (Table 5, *p* = 0.11). Folate deficiency rates decreased in all groups throughout treatment with non-significant differences between groups (Table 4, *p* = 0.15).

Controlling for CRP, serum retinol concentrations decreased slightly in all groups from enrollment to week 10, with non-significant differences between groups (Table 5). Change in percentage deficient or percent with marginal status did not differ significantly by treatment group (Table 4). HIV-positive women had a lower mean retinol concentration (0.96 μmol/L) compared to HIV-negative women (1.12 μmol/L, *p* = 0.007).

Mean serum ferritin concentrations at baseline and at week 10 were similar between groups; all groups had a decrease in ferritin concentrations throughout treatment. Ferritin concentrations at enrollment were significantly related to CRP concentrations, parity, and HIV status. Higher ferritin concentrations were correlated with higher CRP concentrations, HIV-negative status, and primiparity. At enrollment, primiparous women had a mean ferritin of 64.4 ng/mL, compared to 39.0 ng/mL in multiparous women (*p* < 0.01), after adjusting for CRP.

All treatment groups experienced moderate rates of zinc deficiency at enrollment and this did not change in any group in spite of treatments providing >100% of the RDA (Table 4). Women who were in a fasted state at time of blood draw had greater zinc concentrations at enrollment (56.9 µg/dL), compared to 50.7 µg/dL in women who were not fasted (*p* < 0.01). Plasma zinc is expected to decrease in a non-fasted state [16]. There was little change in mean zinc concentration after 10 weeks of treatment, controlling for CRP and fasting status (Table 5). Mean plasma zinc was higher in HIV-negative mothers (55.5 μg/dL, 52.4 μg/dL in HIV+ and HIV−, respectively, *p* = 0.033).

Mean serum albumin concentrations were low across all groups at enrollment and there was no difference in mean serum albumin after 10 weeks of treatment by group (Table 5). Mean serum CRP was elevated at both enrollment and week 10, and was significantly correlated with retinol (*p* < 0.001), ferritin (*p* < 0.001), zinc (*p* = 0.027), and albumin (*p* = 0.004) concentrations. At enrollment, HIV-infected women had a 1.43 times higher geomean CRP than those without HIV (7.54 mg/L vs. 5.27 mg/L, *p* = 0.03). Women who reported having malaria within the previous two months had a 1.9 times greater geomean CRP (15.31 mg/L) compared to women who did not have recent malaria (7.99 mg/L, *p* < 0.001).

Birth length (BL) was correlated with maternal folate and retinol status. A two-fold increase in maternal serum folate over the 10-week period was associated with a 0.54 cm increase in BL (*p* = 0.02). However, BL was negatively correlated with change in serum retinol. Each 0.1 µmol/L increase in serum retinol over 10 weeks was associated with a 0.135 cm decrease in BL (*p* < 0.01). There was no association between trimester or estimated gestational age at enrollment and the effect of retinol on BL, and no association between BL and micronutrient concentrations at enrollment or week 10.

## 4. Discussion

This study tested the hypothesis that providing moderately malnourished pregnant women with supplemental food and micronutrients would improve micronutrient concentrations, and reduce rates of micronutrient deficiencies. All treatment groups contained similar caloric and protein levels, with some variations in micronutrient content (Table 1). After supplementation for 10 weeks, the women in this sub-study did not have improvements in most micronutrients, except vitamins D and B_12_, which had the greatest improvements in the RUSF group. Birth length (BL) was positively correlated with greater maternal serum folate and negatively correlated with greater serum retinol.

Vitamin B_12_ deficiency was found in 20.9% of women at enrollment. Similarly, Sukumar, et al. (2016) found that the average vitamin B_12_ insufficiency from 57 studies was 21%, 19%, and 29% in the first, second, and third trimesters, respectively [14]. Vitamin B_12_ concentrations are known to decrease throughout pregnancy due to a trans-placental transfer of the vitamin [13]. The RUSF and CSB+UNIMMAP groups experienced the smallest decrease in vitamin B_12_ from enrollment to week 10 at 3–8%, compared to a decrease of 20% in the CSB+IFA group, consistent with these two treatments providing higher vitamin B_12_ content at >200% of the RDA. The CSB+IFA group had more than doubled the prevalence of vitamin B_12_ deficiency compared to the RUSF group at week 10. Perhaps the improved serum vitamin B_12_ in the RUSF group was partially due to the vitamin B_12_ in the treatment being in a more bioavailable form due to the presence of milk proteins or other molecules that were not in the CSB+UNIMMAP or CSB+IFA treatment foods. Moreover, lipids in the RUSF treatment may have enhanced bile acid release and uptake of vitamin B_12._ Together, these may have contributed to an improved serum vitamin B_12_ status in the RUSF group following treatment. A study in urban South India found that supplementing pregnant women with 50 µg vitamin B_12_ per day (well above the RDA of 2.6 µg/day) starting at <14 weeks gestation and continuing postpartum resulted in plasma vitamin B_12_ increasing in the second trimester and decreasing by 14% from the second to the third trimester [17], which is much greater than the 3% or 8% decrease found in the RUSF and CSB+UNIMMAP groups, respectively. A Nepalese study of pregnant women found that those with vitamin B_12_ deficiency had a two-fold increased likelihood of preeclampsia and preterm delivery [18]. Maternal vitamin B_12_ deficiency is linked to poor fetal growth and brain development, LBW, and SGA infants [14,19]. This sub-study was not powered to investigate the effect of micronutrients on these birth outcomes.

Only three percent of women in this study were severely vitamin D deficient at enrollment, while marginal status affected one-third of women. Although all treatments contained approximately double the RDA for vitamin D during pregnancy, the RUSF group provided 61 g total fat, 2.26 g of alpha-linolenic acid and 13.96 g of linoleic acid daily. This may have facilitated absorption of vitamin D, as shown by the highest serum vitamin D concentrations in the RUSF group, especially considering dietary fat intake is low in the typical Malawian diet [20]. The higher vitamin D concentrations in the Ngabu clinic were expected, due to the clinic being in a rural area in the valley with higher temperatures and less shade than other districts. It was unexpected to find that HIV-positive women had higher vitamin D concentrations at enrollment. It is possible that women with HIV have differences in diet or sun exposure, but the reasons for these findings are unknown. Inadequate prenatal vitamin D has been linked to adverse birth outcomes, including LBW, premature, and SGA infants [21,22,23]. A recent systematic review and meta-analysis of 24 trials found that prenatal vitamin D supplementation was associated with a reduced risk of SGA as well as increased birth weight in infants whose mothers received vitamin D supplementation after 20 weeks gestation. The authors state that for every 18 pregnant women taking vitamin D supplements, 1 SGA case could be avoided [24]. The increase in the RUSF group of 6.4 ng/mL throughout the present sub-study could be clinically significant in preventing SGA infants or promoting higher birth weights in infants, although the sub-study was not powered to investigate these outcomes. In the larger primary study, the birth length of infants of mothers receiving RUSF was no different than that in the other two treatment groups.

Women in all groups were responsive to folate supplementation, as shown by higher mean serum folate and lower deficiency prevalence in all groups at week 10; rates of deficiency were lowest in the CSB+IFA group at week 10, non-significantly (Table 4). Women with an increase in serum folate had infants with greater BL. A study in China found that women with the lowest serum folate levels were more likely to have infants born with fetal growth restriction [25]. Similar results were found in India, in which pregnant women with greater erythrocyte folate at 28 weeks had infants with greater birth weight [26].

Surprisingly, there was no improvement in serum retinol in spite of provision of >300% of the RDA. The relationship between change in serum retinol and BL was unexpected. In Indonesia, supplementing pregnant women with zinc or vitamin A alone resulted in increased BL by 0.3 cm and 0.2 cm respectively, compared to supplementing vitamin A in combination with zinc (*p* = 0.04) [27]. Higher retinol at 28 weeks (median 1.23 µmol/L), but not at 16 weeks, was associated with lower birth weight (*p* <0.001) in a cohort of pregnant women [28]. In contrast, in a different study, pregnant women receiving MMN supplements (including vitamin A) had infants with 0.80 cm greater BL compared to women receiving IFA alone [29]. A trial with 13,709 infants found no differences in birth size between infants born to mothers receiving vitamin A, beta-carotene, or a placebo [30]. More research is needed to determine if trimester of vitamin A supplementation has an effect on fetal growth. Due to tight homeostatic control, serum retinol may not be the most accurate method for assessing vitamin A status [31,32]. It’s possible that low protein status in this population, as measured by serum albumin, compromised retinol-binding protein synthesis, thus preventing supplemental vitamin A from improving serum retinol concentrations [33]. High amounts of vitamin A were provided in all treatment groups. Since low vitamin A status and inadequate intake of vitamin A rich foods had been identified in previous studies in Malawi, higher than RDA doses were used to improve vitamin A status in this undernourished population [34,35,36]. In a Cochrane review, McCauley et al. (2015) found that pregnant women supplemented with 5000–10,000 IU daily (equivalent to 1500–3000 µg) saw no differences in perinatal mortality or preterm birth compared to pregnant women receiving a placebo [37]. According to the Cochrane review and the WHO, daily doses of 3000 µg retinol after day 60 of gestation are likely safe, especially in vitamin A deficiency endemic areas. Beyond 60 days gestation, the risk of teratogenicity with vitamin A supplementation >3000 µg diminishes [38]. Undernourished women in this study enrolled in their second trimester, well after the first 60 days of gestation. The vitamin A UL was exceeded slightly in the CSB+UNIMMAP group due to the use of the standard CSB+ formulation combined with the standard UNIMMAP content. This treatment option was included since it would be a simple intervention using well-known and available treatments and would be less expensive than RUSF.

Despite receiving 45–79 mg iron daily and controlling for the presence of inflammation using CRP, ferritin concentrations were lower in all treatment groups at the 10-week measurement compared to concentrations at enrollment, and the prevalence of deficiency increased similarly in all groups. Iron deficiency was common, whether providing ~170% (CSB+UNIMMAP or RUSF) or 292% (CSB+IFA) of the RDA for iron. The CSB+IFA group received 79 mg iron daily, due to the CSB+ formulations by the World Food Program combined with the supplemental amount of iron in the standard of care for pregnant women [39]. Thus, amounts of iron greater than 45 mg do not appear beneficial in improving ferritin levels during pregnancy. However, serum ferritin tends to decrease between weeks 12 and 25 of gestation due to expansion of maternal red blood cell mass [40,41]. In this study, primiparous women had higher ferritin concentrations at enrollment. Additional pregnancies are likely to result in the mother becoming nutritionally depleted, especially if they are closely spaced [41]. Although ferritin did not have significant improvements in any treatment group, it is possible there were changes in iron transport or metabolism, although we do not have data on other iron biomarkers, such as transferrin.

Maternal plasma zinc declines during pregnancy and dietary zinc intake in Malawian women has been reported as inadequate [42,43]. At week 10, there was no change in plasma zinc in any group while controlling for inflammation; approximately one-third of women remained zinc deficient after supplementation with up to 243% the RDA of zinc, suggesting that other factors are contributing to deficiency. A potential factor in the iron and zinc concentrations of most women not improving despite the treatments containing well above the RDA for both minerals (zinc in CSB+IFA being the exception) could be the presence of phytates in the study foods, as phytates inhibit absorption of minerals, including iron, zinc, and calcium. Cereals and grains are staples in the Malawian diet, which are both low in zinc and high in phytates. Phytates are also found in corn, the basis of the CSB+ supplementary food. Considering that the presence of vitamin C typically enhances iron uptake, the lack of iron improvement despite high vitamin C content may be due to the dietary phytates in the treatment foods. Another potential factor is that the relatively high calcium content in the treatments may have attenuated improvement of mineral status by inhibiting iron and zinc absorption.

Mean serum albumin in all groups, controlling for inflammation, was low at baseline, with no increase in any group, which is not surprising since all treatments contained comparable amounts of protein. A similar study found that pregnant Malawian women at a mean gestational age of 24 weeks had mean serum albumin concentrations of 3.1 ± 5 mg/L [43], which is slightly lower than in the present study. CRP concentrations in this population were highly elevated, even in the absence of HIV or self-reported malaria, pneumonia, or diarrhea, and considering inflammation associated with pregnancy, indicating that there may be other underlying causes of inflammation. A study in Ghanian pregnant women found that mean CRP concentrations were 5.8–7.9 mg/L [11], consistent with the results in the present sub-study. Future research could consider the impact of parasites or other microbiota-disrupting pathogens that could impact inflammatory status.

Inflammation that occurs during the acute phase response is known to alter absorption and serum or plasma concentrations of many nutrients, including retinol, ferritin, and zinc, thus we used CRP as a controlling measure in statistical analyses for these nutrients. Retinol and plasma zinc concentrations are reduced during an inflammatory response, while ferritin concentrations will increase [15,44]. The effect that inflammation has on retinol levels depends largely on the individual’s overall nutritional status. Inflammation will result in reduced levels of circulating free iron, while increasing binding and storage proteins such as ferritin [44].

In the primary Mamachiponde study, almost 20% of infants were born at low birth weight and more than 21% were born stunted [12]. Maternal deficiencies in vitamin D [23], vitamin B_12_ [45], iron [46] and zinc [6] have been linked to LBW infants, indicating that single or multiple micronutrient deficiencies in the women in this study may play a role in the lower birth weights observed overall in the primary study. Since the risks of poor outcomes are considerable for each deficiency, further research is needed on the appropriate supplemental micronutrient dose for pregnant women with moderate malnutrition and how to target and deliver supplements to such women.

Limitations include the inability to double blind the study due to visible differences between interventions. Loss to follow-up was high (*n* = 112, 33%). The primary reason for the high loss to follow-up was due to women delivering their baby before the 10-week treatment period ended. In the primary study, average gestational age at enrollment was 19 weeks and average gestational age at delivery was at 31 weeks, leaving a small window for measurement of supplementation effect. We attempted to enroll women in the study as early as possible, but they typically did not attend antenatal clinic until the second trimester, thus 10 weeks was about the longest period of treatment time that was possible without risking higher loss to follow-up. In the primary Mamachiponde study, mean time from enrollment to delivery was approximately 11 weeks. A longer period of exposure to treatment most likely would have improved serum concentrations and decreased rates of deficiency, however, this was not possible. The prevalence of folate and vitamin D deficiencies did decrease; therefore it appears 10 weeks was adequate time to respond to the supplementation for these nutrients, but perhaps not the others, especially considering the metabolic demands of pregnancy. In addition, recovery from a nutrient deficiency can also be affected by the level of deficiency and the amount of the nutrient provided in the supplementation regimen. Therefore, it is difficult to determine which specific factor (duration of supplementation, amount of nutrients provided, level of deficiency of subject) led to limited repletion in this study. These may be potential considerations for future studies using a similar intervention. Another limitation included fasting status. Due to women having to walk far distances to the clinic, it was unethical to advise women to fast before their blood draws. Sixty-six percent of women were fasted for the first blood draw; 37% were fasted for the second blood draw. Zinc concentrations were affected by fasting status, which was controlled for in analysis. Only fundal height measurements were used to estimate gestational age due to lack of ultrasound equipment, thus reducing accuracy of reported gestational age. This study only included pregnant women with moderate acute malnutrition, thus it does not provide information concerning the population of all pregnancies.

## 5. Conclusions

In conclusion, more than 20% of pregnant women with moderate acute malnutrition had one or more micronutrient deficiencies and supplementation with 200% or more of the RDA of most micronutrients did not improve their nutrient status, with the exception of vitamin B_12_ and vitamin D showing the most improvement in the RUSF group. Although only two of the nutrients measured saw a benefit from the RUSF treatment, it is clear that the current antenatal standard of care (CSB+IFA) is not enough to improve the nutritional statuses of moderately malnourished pregnant women. More research should be conducted to determine the best proportions of supplementary micro- and macronutrients to help pregnant women recover from nutritional deficiencies and improve maternal and infant outcomes. A supplementary food containing lipids should be considered to improve absorption and utilization of nutrients in the body. However, since most micronutrients did not improve, this indicates that other factors are altering their nutritional state, potentially including infection, inflammation, malabsorption, genetic differences, or nutrient interactions that play a role in determining nutrient status.

## Figures and Tables

**Table 1:** Nutrient comparison by treatment group ^1^.

Nutrient	CSB+IFA ^2^	CSB+UNIMMAP ^3^	RUSF ^4^	RDA ^5^	UL ^6^
Energy (kcal)	893	893	920	NA	NA
Protein (g)	33	33	36	NA	NA
α-linolenic acid (g)	0	0	2.3 (161)	1.4	NA
Linoleic acid (g)	0	0	14.0 (107)	13	NA
Docosahexaenoic acid (mg)	0	0	211	NA	NA
Eicosapentaenoic acid (mg)	0	0	43	NA	NA
Vitamin A (µg)	2410 (312)	3210 (417)	2628 (341)	770	3000
Vitamin B_1_ (mg)	0.3 (20)	1.7 (121.5)	3.2 (228)	1.4	NA
Vitamin B_2_ (mg)	3.29 (235)	4.7 (355)	3.8 (270)	1.4	NA
Vitamin B_3_ (mg)	18.8 (104)	36.8 (204)	35 (194)	18	35
Vitamin B_6_ (mg)	4.0 (210)	5.9 (310)	4.0 (210)	1.9	100
Vitamin B_12_ (µg)	4.7 (181)	7.3 (280)	5.5 (211)	2.6	NA
Folic acid (µg)	659 (165)	659 (165)	574 (143)	400	1000
Vitamin C (mg)	211 (249)	281 (331)	170 (200)	85	2000
Vitamin D (µg)	25.4 (169)	20.9 (139)	30 (200)	15	100
Vitamin E (mg)	19.5 (130)	29.5 (197)	39.2 (261)	15	1000
Vitamin K (µg)	70.5 (78)	70.5 (78)	192 (213)	90 ^7^	NA
Iodine (µg)	94 (43)	244 (111)	300 (136.4)	220	1100
Copper (µg)	0	2000 (200)	2400 (240)	1.0	10.0
Iron (mg)	79.2 (292)	45.3 (180.7)	45 (170)	27	45
Zinc (mg)	11.8 (107)	26.8 (243)	24.6 (223)	11	40
Magnesium (mg)	400 (114)	400 (114)	327 (93)	350	350
Calcium (mg)	851 (85)	851 (85)	1830 (183)	1000	2500
Selenium (µg)	0	65 (108)	123 (205)	60	400

^1^ Values expressed as amounts (% RDA). RDA: Recommended Dietary Allowance; CSB+: Fortified corn-soy blend; IFA: Iron and folic acid; NA: Not applicable; RUSF: Ready-to-use supplementary food; UNIMMAP: UN International Multiple Micronutrient Preparation; ^2^ Estimates a daily portion of 235 g CSB+ per day plus iron (60 mg) and folic acid (400 µg); ^3^ Estimates a daily portion of 235 g CSB+ per day plus UNIMMAP supplement; ^4^ Estimates a daily portion of 175 g RUSF per day; ^5^ RDA for pregnant women aged 19–30 years; ^6^ UL: Tolerable upper limit for pregnant women aged 19–30 years; ^7^ Indicates Adequate Intake (AI) rather than RDA.

**Table 2:** Deficiency and marginal status cut-off values for pregnancy and inflammatory states.

7.5	Units	Severely Deficient	Deficiency	Deficiency with Inflammation	Marginal Cut-off Values
Vitamin B_12_^1^	pg/mL		<203.0	--	--
Vitamin D ^2^	ng/mL	<12.0	12–20	--	20–30
Folate ^3^	ng/mL		<3.0	--	3.0–5.9
Retinol	µmol/L		<0.70	<0.60 ^4^	<1.05
Ferritin	ng/mL		<15.0	<19.0 ^4^	--
Zinc ^5^	µg/dL		1st trimester: <57 2nd/3rdtrimester: <50	--	--
Albumin	g/dL		<3.4	--	--
CRP ^6^	mg/L		Elevated >5.0	--	--

^1^ Vitamin B_12_ cut-off value determined from [13,14]; ^2^ Vitamin D cut-off values determined using Associated Regional and University Pathologists (ARUP) Laboratory Reference Ranges; ^3^ Folate cut-off values determined from [13,15]; ^4^ Retinol and ferritin inflammation cut-off values determined from [15]; ^5^ Zinc deficiency cut-off values for pregnancy determined from [16]; ^6^ CRP (C-reactive protein) elevation cut-off value determined from [15].

**Table 3:** Selected characteristics of the participants at enrollment, by treatment group.

Characteristic	CSB+IFA (*n* = 115)	CSB+UNIMMAP (*n* = 110)	RUSF (*n* = 118)	*p* ^1^
Age at enrollment (years)	20.8 ± 4.4 ^2^	20.6 ± 3.8	20.4 ± 4.7	0.79
16–17 (%)	19.1	13.6	26.2	0.15
18–21 (%)	52.2	58.2	44.1	
22–29 (%)	19.1	23.6	22.9	
30–40 (%)	9.6	4.6	6.8	
Illness (previous 2 months, %)	22.6	21.1	20.3	0.92
Diarrhea (previous 2 months, %)	5.2	6.5	4.2	0.75
Malaria (previous 2 months, %)	13.9	13.0	17.8	0.55
Fundal height (cm)	19.7 ± 3.6	19.6 ± 4.2	18.4 ± 4.4	0.04
Previous pregnancies	1.0 ± 1.3	1.0 ± 1.3	0.9 ± 1.6	0.84
Currently taking supplements	67.8	70.6	72.0	0.78
Folic acid (%)	3.5	0.9	2.5	0.43
Iron (%)	64.4	68.2	69.5	0.69
HIV status (% positive) ^3^	15.8	9.2	8.6	0.16
Height (cm)	154.1 ± 5.4	153.9 ± 5.3	154.2 ± 5.3	0.92
MUAC ^4^ (cm)	22.2 ± 0.6	22.2 ± 0.6	22.2 ± 0.6	0.84
Weight (kg)	45.7 ± 3.5	45.8 ± 3.8	45.9 ± 3.8	0.92
BMI ^5^ (kg/m^2^)	19.2 ± 1.1	19.3 ± 1.3	19.3 ± 1.4	0.80
Underweight (%) ^6^	30.4	25.5	28.8	0.70

^1^*p*-values obtained by ANOVA for continuous variables and chi-squared test for proportions; ^2^ Mean ± SD (for all such values unless noted otherwise); ^3^ HIV: Human Immunodeficiency Virus; ^4^ MUAC: Mid-upper arm circumference; ^5^ BMI: Body Mass Index; ^6^ Underweight: BMI <18.5 kg/m^2^

**Table 4:** Prevalence of micronutrient deficiency by treatment group, at week 0 and week 10.

Nutrient	CSB+IFA ^1^		CSB+UNIMMAP ^2^		RUSF ^3^		*p* ^4^
	Week 0	Week 10	Week 0	Week 10	Week 0	Week 10	
Vitamin B_12_^5^	18.0	41.8	22.0	23.0	22.6	20.5	<0.001
Vitamin D ^6^							
Marginal	34.2	24.1	33.0	24.3	38.3	23.1	0.98
Deficient	1.8	0.0	0.9	1.4	7.0	2.6	
Folate ^7^	26.1	6.3	20.2	16.2	18.3	12.8	0.15
Retinol							
marginal ^8^	34.9	25.3	37.0	32.4	25.9	33.3	0.49
deficient ^9^	4.6	7.6	8.3	8.1	6.0	5.1	0.74
Ferritin ^10^	25.2	48.7	26.6	42.5	23.5	53.8	0.37
Zinc ^11^	24.8	32.9	16.8	33.8	25.0	29.9	0.86

^1^ CSB + IFA: Corn-soy blend with iron and folic acid supplement; ^2^: CSB + UNIMMAP: Corn-soy blend with UN International Multiple Micronutrient Preparation; ^3^ RUSF: Ready-to-use supplementary food; ^4^ Proportions of deficiency and *p*-value determined using chi-square testing for categorical variables; *p*-value indicating differences between treatment groups at week 10; ^5^ Cutoff for B_12_ deficiency <203 pg/mL; see Table 2 for references on all deficiency cut-off points; ^6^ Cutoff for vitamin D deficiency <20 ng/mL, marginal or deficient <30 ng/mL; ^7^ Cut off for marginal folate status <5.9 ng/mL; ^8^ Cutoff for marginal retinol status adjusting for inflammation using <0.93 µmol/L; ^9^ Cutoff for retinol deficiency adjusting for inflammation <0.60 µmol/L; ^10^ Cutoff for ferritin deficiency adjusting for inflammation using <19 ng/mL; ^11^ Cutoff for zinc deficiency in 1st trimester: <57 µg/dL; 2nd or 3rd trimesters: <50 µg/dL.

**Table 5:** Adjusted ^1^ mean (SE) or geomean serum/plasma ^2^ concentrations by treatment group at week 0 and week 10.

Outcome	CSB+IFA ^3^		CSB+UNIMMAP ^4^		RUSF ^5^		*p* ^6^
	Week 0	Week 10	Week 0	Week 10	Week 0	Week 10	
	(*n* = 111)	(*n* = 79)	(*n* = 109)	(*n* = 74)	(*n* = 115)	(*n* = 78)	
Vitamin B_12_ (pg/mL) ^7,10^	281 ^b^	225 ^a^	277 ^a^	255 ^a,b^	285 ^a^	275 ^a^	0.001
	Δ = 0.80 ^a^		Δ = 0.92 ^b^		Δ = 0.97 ^b^		
25-hydroxy-vitamin D (ng/mL) ^11^	34.6 (0.9) ^c^	36.3 (1.0) ^b,c^	35.9 (0.9) ^c^	38.6 (1.0) ^a,b^	34.5 (0.9) ^c^	40.9 (1.0) ^a^	<0.001
	Δ = 1.7 (0.8) ^a^		Δ = 2.7 (0.8) ^a^		Δ = 6.4 (0.8) ^b^		
Folate (ng/mL)	8.9 (0.5)	13.1 (0.6)	9.7 (0.6)	11.7 (0.7)	9.4 (0.6)	11.6 (0.7)	0.11
Retinol (μmol/L) ^8^	1.05 (0.04)	1.01 (0.04)	1.07 (0.04)	1.05 (0.04)	1.05 (0.04)	1.01 (0.04)	0.93
Ferritin (ng/mL) ^7,10^	29.1	18.6	29.0	20.9	33.2	18.2	0.30
Plasma zinc (μg/dL) ^8,9^	53.8 (1.1)	53.1 (1.2)	55.0 (1.1)	53.8 (1.3)	53.1 (1.1)	54.9 (1.3)	0.40
Albumin (g/dL) ^8^	3.44 (0.05)	3.27 (0.05)	3.41 (0.05)	3.29 (0.06)	3.48 (0.05)	3.19 (0.06)	0.43
C-reactive protein (mg/L) ^7^	6.5	6.1	7.0	6.9	7.3	5.2	0.51

Values in a row with differing superscript letters indicate significant differences between groups, based on Tukey-Kramer adjustments; ^1^ Adjusted for enrollment MUAC, HIV status, parity, and fundal height; ^2^ All nutrients were assessed using serum with the exception of plasma zinc; ^3^ CSB + IFA: Corn-soy blend with iron and folic acid supplement; ^4^ CSB+UNIMMAP: Corn-soy blend with UN International Multiple Micronutrient Preparation; ^5 ^ RUSF: Ready-to-use supplementary food; ^6^ *p*-value attained by RMANOVA; *p*-value partial F-test for treatment effect (main effect or time interaction); ^7^ Logarithmic transformation used. Values are geomeans; ^8^ Additionally adjusted for log(CRP) concentration; ^9^ Additionally adjusted for fasting status; ^10^ Δ:Multiplicative change from week 0 to week 10; ^11^ Δ:The week 0 to week 10 difference.

## Supplementary Materials

The following are available online at http://www.mdpi.com/2072-6643/10/7/879/s1, Figure S1: Participant Flowchart, Table S1: Assays used for analysis of nutrients and proteins.
