# Supplementary material for: Effect of Nutritional Interventions on Micronutrient Status in Pregnant Malawian Women with Moderate Malnutrition: A Randomized, Controlled Trial
Source: Nutrients. 2018 Jul 7;10(7):879. doi: 10.3390/nu10070879 (PMC6073606; doi:10.3390/nu10070879)
Supplement: Supplementary file 1 [file nutrients-10-00879-s001.pdf]

Supplemental Figure 1

Participant Flowchart

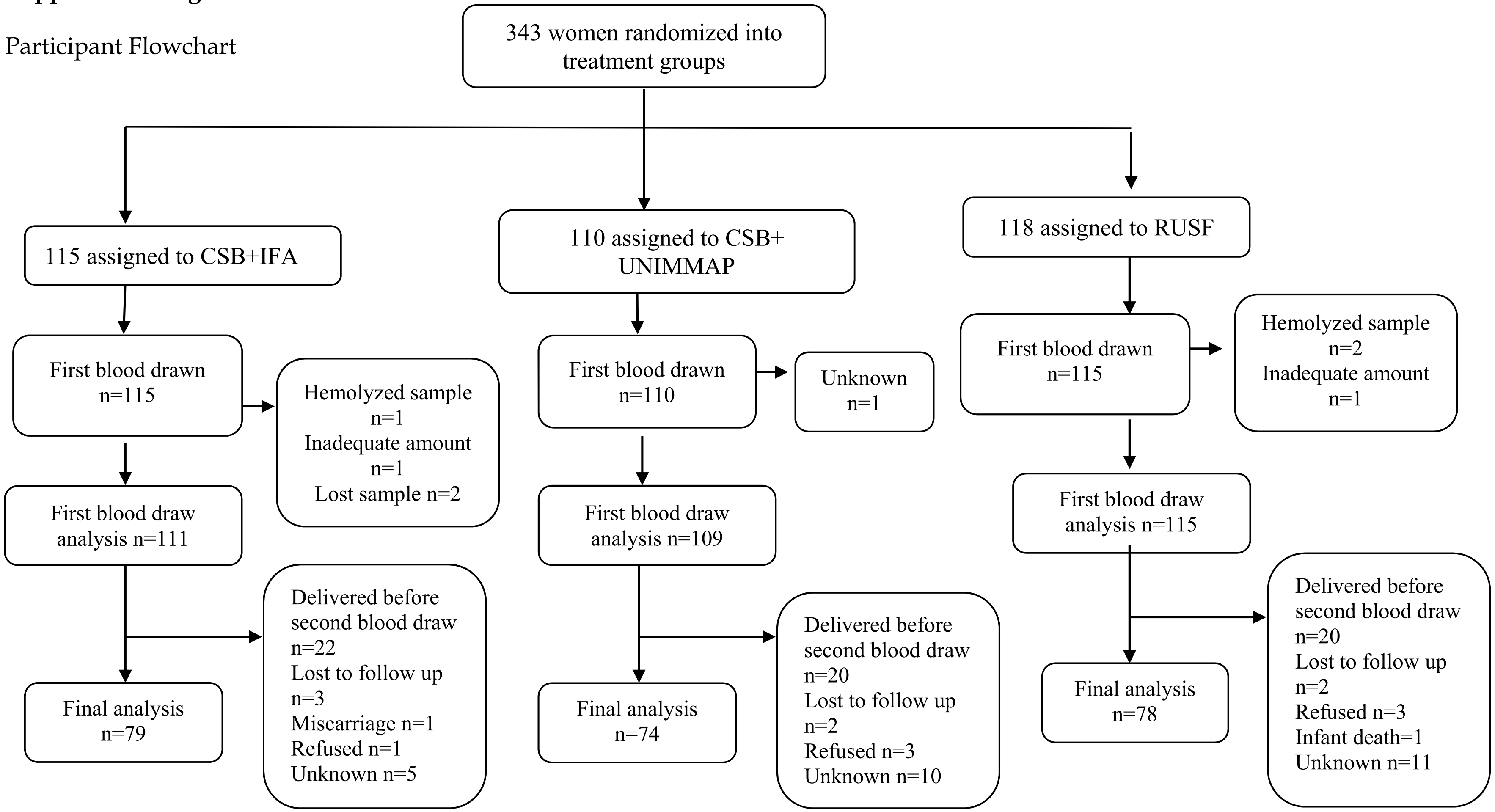

Supplemental Table 1
Assays used for analysis of nutrients and proteins

Nutrient/protein	Assay method- CCP[1]	Assay method-PAL[2]
Vitamin B_{12}	ECLIA[3]	Chemiluminescence (Beckman Coulter DXI)[4]
25-hydroxyvitamin D	N/A	Chemiluminescence[5] (Liason XL Diasorin)
Folate	ECLIA	N/A
Retinol	Nephelometry[6]	Chemiluminescence
Ferritin	Electrochemiluminescence Immunoassay (ECLIA)[7]	Chemiluminescence (Beckman Coulter DXI)
Zinc	Quantitative Inductively Coupled Plasma-Mass Spectrometry[8]	Plasma-Mass Spectrometry
Albumin	Bromcresol Green (BCG)[9]	BCG (Beckman Coulter DXI)
C-reactive protein	Immunoturbidimetry[10]	Immunoturbidimetry (Beckman Coulter 5800)

[1]Central Coast Pathology
[2]Physician's Automated Laboratory
[3]Pirkle JL. "Laboratory Procedure Manual. Vitamin B12 Roche E-170 Vitamin B12 ECLIA." *CDC* (2012).
[4] Kumar SS, Chouhan RS, Thakur NS. "Enhancement of Chemiluminescence for Vitamin B12 Analysis." *Anal Biochem* 388.2 (2009): 312–6.
[5] Wootton AM. "Improving the Measurement of 25-Hydroxyvitamin D." *Clin Biochem Rev* 26.1 (2005): 33–36.
[6] dePee S and Dary O. "Biochemical Indicators of Vitamin A Deficiency : Serum Retinol and Serum Retinol Binding Protein." *J Nutr* 132 (2002): 2895S–2901S.
[7] Dupuy, AM, L Debarge, et al. "Determination of Serum Ferritin Using Immunoturbidimetry or Chemiluminescent Detection in Comparison with Radioimmunoassay a Compendium of a Methodological Juxtaposition." *Clin Lab* 55.5–6 (2009): 207–15.
[8] Vanhoe, H et al. "Determination of Iron, Cobalt, Copper, Zinc, Rubidium, Molybdenum, and Cesium in Human Serum by Inductively Coupled Plasma Mass Spectrometry." *Anal Chem* 61.17 (1989): 1851–57.
[9] McGinlay, JM, and RB Payne. "Serum Albumin by Dye-Binding: Bromoscresol Green or Bromocresol

Purple? The Case for Conservatism." *Ann Clin Biochem* 25.4 (1988): 417–21.
[10] Dupuy, AM, S Badiou, et al. "Immunoturbidimetric Determination of C-Reactive Protein (CRP) and High-Sensitivity CRP on Heparin Plasma. Comparison with Serum Determination." *Clin Chem Lab Med* 41.7 (2003): 948–9.
